# Development and Validation of Epilepsy Life Skills Guidelines for Primary School Learners and Teachers in Limpopo and Mpumalanga Provinces

**DOI:** 10.3390/children10071194

**Published:** 2023-07-10

**Authors:** Thendo Gertie Makhado, Rachel Tsakani Lebese, Maria Sonto Maputle

**Affiliations:** 1 Department of Advanced Nursing Sciences, Faculty of Health Sciences, University of Venda, P. Bag X5050, Thohoyandou 0950, South Africa; sonto.maputle@univen.ac.za; 2 The Research Office, Faculty of Health Sciences, University of Venda, P. Bag X5050, Thohoyandou 0950, South Africa; rachel.lebese@univen.ac.za

**Keywords:** development, epilepsy, educational program, guidelines, life skills, primary school learners

## Abstract

Epilepsy is a disorder that disturbs nerve cell activity in the brain, resulting in seizures. It was noted that it occurs mostly in children, especially at the primary school level, and could result from a genetic condition. The aim of this study was to develop and validate life skills education guidelines for primary school learners of Limpopo and Mpumalanga provinces in South Africa to educate them about epilepsy with the aim of reducing stigma and discrimination towards people with epilepsy. The guidelines were developed by adapting the World Health Organization (WHO) guideline development guide, which included the formulation of PICOS (population, intervention, comparison, outcome, study design) questions, conducting a systematic review of the literature, and using GRADE (grading of recommendations assessment, development, and evaluation) to develop evidence-based recommendations. The recommendations that informed the guideline development were that epilepsy education should be included in the primary school life skills curriculum to improve learners’ understanding, attitudes, and skills related to epilepsy. This epilepsy education should be tailored to the needs of primary school learners and cover topics such as seizure recognition, management, medication, and coping strategies. Collaboration between healthcare providers, educators, and policymakers is crucial to developing culturally appropriate and evidence-based epilepsy education guidelines. Teachers and healthcare providers should also receive training on how to support learners living with epilepsy. The resulting guidelines provide clear and concise guidance on epilepsy education in life skills for primary school learners, caregivers, and teachers. The guidelines are expected to improve the quality of epilepsy life skills education and contribute to the overall well-being and inclusion of learners with epilepsy in Mpumalanga and Limpopo provinces, South Africa.

## 1. Introduction

People of all ages, including children, are impacted by the neurological condition known as epilepsy. Aaberg [1] reports that 1 in 150 children is diagnosed with epilepsy internationally, while Thijis [2] estimates that 50 million people worldwide suffer from the ailment. According to Lystad [3], children with epilepsy experience unique difficulties, especially in their social and academic lives. These difficulties might severely impact their general well-being and academic success. Studies have shown that people living with epilepsy (PLWE) are more likely to experience social isolation, reduced opportunities for employment and education, increased psychological distress, and anxiety [4,5]. Stigma and discrimination can also lead to delays in seeking medical care and treatment and reduced adherence to treatment regimens [6,7]. Although there is no known cure for epilepsy, effective management of the condition can significantly enhance the quality of life of PLWE.

Studies revealed that epilepsy is given less attention compared to other chronic conditions in terms of education, and this condition is associated with stigma, misconceptions, and discrimination that are contributing to school dropouts [8,9]. Since there is a high incidence of epilepsy in sub-Saharan Africa (SSA), one study conducted in SSA revealed that learners living with epilepsy were dropping out and sometimes missing classes due to perceived stigma from classmates [10]. This means there is a necessity to bridge the gap of a lack of epilepsy education starting from primary schools to mitigate stigma related to epilepsy from a young age.

Several countries, such as India, Bolivia, and Pakistan, have addressed the stigma and discrimination associated with epilepsy through various initiatives, including educational programs and public awareness campaigns [11,12,13]. Recent research in South Africa has suggested that epilepsy should be covered in the primary school life skills curriculum. Doing so may help educate learners about the condition at a young age and lessen stigma, misunderstandings, and discrimination [14]. Additionally, it was found that teaching epilepsy in schools may help spread knowledge and awareness to the broader community through learners, potentially reducing misconceptions and stigma related to epilepsy [14]. 

The prevalence of stigma and discrimination associated with chronic illnesses, such as HIV, mental illness, and diabetes mellitus, has led to the development of educational programs aimed at reducing these negative attitudes. Studies suggest that insufficient knowledge about the nature of these conditions contributes to stigmatization [15,16,17]. It is evident that these educational interventions decreased the stigma and discrimination surrounding these chronic conditions [17]. Considering this, it is hypothesized that the development and implementation of epilepsy life skills guidelines may be effective in mitigating stigma and discrimination associated with this particular chronic illness, as has been demonstrated in the case of other chronic conditions.

This research paper aims to establish guidelines for epilepsy life skills education specifically designed for primary school learners and teachers in the Limpopo and Mpumalanga provinces. To develop these guidelines, the researchers adapted the World Health Organization’s guidelines guide [18], which incorporates the population, intervention, comparison, outcome, and study design (PICOS) framework and the grading of recommendations assessment, development, and evaluation (GRADE) methodology.

## 2. Materials and Methods

This study is a component of the third phase of a broader research project that aims to create life skills education guidelines for epilepsy in primary schools in Limpopo and Mpumalanga provinces. The primary objective of these guidelines is to mitigate the stigmatization and misconceptions surrounding epilepsy within the community. Phase 1 employed a qualitative multimethod research approach, with stages 1 and 2 of the empirical phase employing an exploratory–descriptive study design. The study focused on the perceptions or views of primary school teachers, life skills educational advisors, and learners regarding the need to include epilepsy in life skills education. The feasibility of the study was assessed through pre-testing to ensure that the central questions were clear enough and understandable.

Data were collected through individual interviews with life skill educational advisors and teachers and focus group discussions with learners in the sampled primary schools in Limpopo and Mpumalanga provinces. Trustworthiness was achieved by enhancing credibility, dependability, conformability, and transferability. The data were analyzed using ATLAS.ti following the basic steps of the notice-collect-think (NCT) analysis. 

The preliminary results included 3 main themes: the advantages of incorporating epilepsy education, the proposed content in epilepsy education, and the recommended teaching methods for integrating epilepsy education into life skills instruction (See Table 1) 

The outcomes of the first two stages were used to develop a conceptual framework in phase 2 and have demonstrated a need for incorporating epilepsy education into life skills instruction. Furthermore, the conceptual framework served as the basis for development of epilepsy life skills guidelines for Limpopo and Mpumalanga provinces.

This paper adapted the WHO [18] guideline development guide to develop guidelines for a specific health intervention. Guideline development is a systematic and rigorous process involving a series of steps to provide evidence-based recommendations to healthcare practitioners and policymakers. The guideline development process typically involves identifying the need for the guidelines, formulating the questions that the guidelines will address, conducting a systematic review of the relevant evidence, and formulating recommendations based on the quality of the evidence. The guideline development process adhered to a systematic approach, detailed as follows.

### 2.1. Identify the Need for the Guidelines and Establish the Guideline Topic

The World Health Organization WHO [18] aims to develop guidelines in an objective and transparent manner, taking into account the best available evidence and stakeholders’ values and preferences. To develop epilepsy life skills guidelines, the researcher conducted interviews with primary school learners, life skills educational advisors, and teachers to determine the need for including epilepsy in life skills education. The results showed that including epilepsy in life skills education is necessary [14,19], and a conceptual framework was developed to assist in the guideline development process. The conceptual framework that was developed highlighted the importance of establishing a strong base centered around individual self-esteem. This can be enhanced through three key elements: educating individuals about epilepsy, exploring their values and attitudes regarding epilepsy, and empowering them to acquire skills and engage in suitable social interactions regarding epilepsy. Furthermore, participants emphasized the importance of using various teaching methods to increase knowledge and understanding of epilepsy, promote positive values and attitudes, and develop skills to manage seizures. Therefore, the guidelines should reflect these aspects and provide guidance on the most effective teaching methods to achieve these objectives.

According to the study’s findings, it is essential to teach primary school learners about epilepsy in life skills to raise awareness of the ailment and eradicate common misconceptions about it. The promoters of the study oversaw the creation of the life skills guidelines, which focused on the topic of “Development and validation of Epilepsy Life Skills Guidelines for Primary School Learners and Teachers in Limpopo and Mpumalanga Provinces”. The guidelines are expected to provide a foundation for teaching epilepsy in life skills education in primary schools located in Mpumalanga and Limpopo provinces. This, in turn, will improve awareness and understanding of epilepsy.

### 2.2. The Scope of the Guidelines

According to WHO [18], scoping of guidelines is the process that involves establishing the specific content and details that will be covered by the guidelines. The researcher determined the scope of the epilepsy life skills guidelines for primary school students and teachers by utilizing the findings from empirical research. The guidelines were designed to target primary school learners in Limpopo and Mpumalanga provinces, focusing on reducing the stigma associated with epilepsy and promoting epilepsy awareness from an early age among primary school learners. The following questions guided the search for data and assisted in formulating potential recommendations.

- What are the benefits of including epilepsy in life skills education for primary school learners?
- What content should be included in epilepsy education for primary school learners?
- What teaching methods would most effectively deliver epilepsy education to primary school learners?

### 2.3. Formulation of Questions

The conceptual framework that was devised provided guidance for the creation of the PICOS model, which seeks to enhance the instruction of epilepsy life skills within primary school settings. The implications of implementing this model encompass various objectives, including increasing knowledge and awareness about epilepsy, reducing social stigma associated with the condition, improving the management of seizures, enhancing support systems for individuals and their families, and advancing the field of epilepsy education. These objectives can be achieved through the implementation of three core components, namely, educating learners about epilepsy, exploring values and attitudes towards epilepsy, and facilitating the development of skills and appropriate social interactions pertaining to epilepsy.

The researcher employed the PICO framework to formulate questions to guide the systematic search for evidence. The PICO framework is a commonly used tool in evidence-based research that helps to clarify the critical components of a research question, including the population of interest, the intervention being studied, the comparator group, the outcomes being measured, and the study design. The PICOS is described hereunder.

#### 2.3.1. Population

The population refers to the individuals or patients who will be the subject of the intervention or the recommended course of action.

The population of interest is primary school learners, which includes both males and females in grades 4–7 aged from 9–11 years. These are the critical years of development for children and are considered an opportune time for imparting knowledge, values, and skills related to epilepsy [21]. The other population of interest is the primary school teachers, including both males and females and those who teach life skills from grades 4–7.

#### 2.3.2. Intervention

This refers to the course of action or therapy that the guidelines will advise upon. The intervention being studied is an epilepsy life skills education program through educating, exploring, and enabling. This program includes teaching strategies to improve knowledge and understanding of epilepsy, values and attitudes towards epilepsy, and skills related to recognizing and responding to seizures.

#### 2.3.3. Comparator

Comparator refers to the alternate therapies or interventions that will be compared to the guideline’s suggested intervention [18]. The comparator group in this study is either normal education or a control group that did not receive the epilepsy life skills education program. Normal education refers to the standard curriculum provided to primary school learners without additional intervention or modification. On the other hand, the control group is a group of primary school learners who did not receive the epilepsy life skills education program but received a placebo or no intervention.

#### 2.3.4. Outcome

This refers to the result or results that which will be evaluated to determine whether the suggested intervention was effective. The outcomes being measured in this study are knowledge and understanding of epilepsy, values and attitudes towards epilepsy, and skills related to recognizing and responding to seizures. These outcomes are important for assessing the effectiveness of the epilepsy life skills education program and its impact on improving the quality of life for primary school learners with epilepsy.

#### 2.3.5. Study Design

Refers to the kind of study design that will be utilized in the review or formulation of guidelines. The review included studies using randomized controlled trials (RCT) or quasi-experimental designs. 

**Figure 1:**
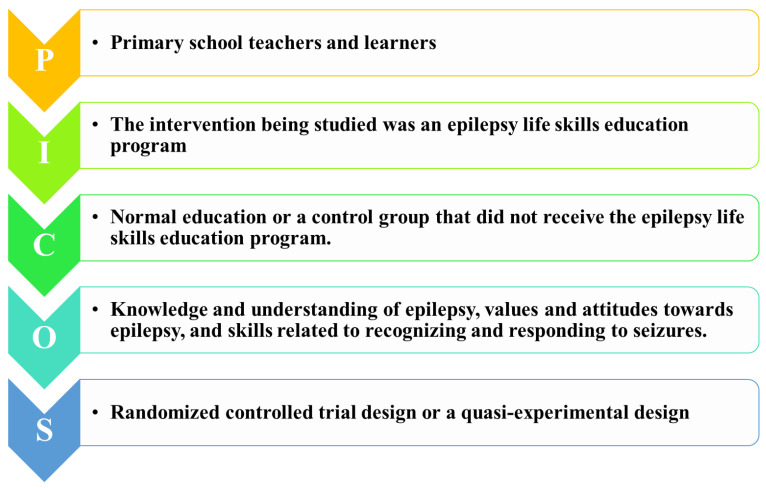
WHO (PICOS) model on formulating the question.

The formulated PICO question was “Among primary school learners and teachers, does an epilepsy life skills education program through educating, exploration, and enabling, compared to normal education or a control group, improve knowledge and understanding of epilepsy, values and attitudes towards epilepsy and skills related to recognizing and responding to seizures?” Figure 1 indicates the PICO model utilized to formulate the question.

### 2.4. Evidence Retrieval and Synthesis

It is imperative to comprehensively review the literature and evidence about the phenomenon being studied to establish a reliable guideline. This assertion is supported by the World Health Organization [18], which underscores the importance of thoroughly investigating the existing body of knowledge related to the research topic. Therefore, the systematic review was conducted based on the PICOS question. The systematic review revealed strong evidence that epilepsy life skills education programs are effective in improving knowledge and understanding of epilepsy [21,22,23,24], and skills related to recognizing and responding to seizures among primary school learners [25]. It was further recommended that healthcare providers, educators, and policymakers should consider incorporating epilepsy life skills education programs into primary school curricula to reduce stigma and improve the quality of life for people living with epilepsy [12,21,22,23,24,25,26,27,28,29]. However, further research is needed to determine these interventions’ most effective content, duration, and delivery method.

### 2.5. Evidence Assessment

When conducting a systematic review to develop guidelines, it is crucial to evaluate the quality of evidence gathered and synthesized. To assess the quality of evidence, the researcher in this study utilized the GRADE (grading of recommendations, assessment, development, and evaluation) approach as recommended by the World Health Organization [18]. This approach involves assessing the quality of evidence and developing recommendations based on the retrieved articles. The GRADE approach categorizes evidence into four levels: high, moderate, low, or very low. 

WHO [18] emphasizes that the quality of evidence depends on the study’s design, with randomized controlled trials being considered high-quality evidence, while evidence from observational studies is given a lower rating. The systematic review included interventional studies (randomized controlled trials) and quasi-experimental studies. When evaluating the quality of evidence using the GRADE approach, five factors are considered: risk of bias, imprecision, directness, consistency, and reporting bias [18]. These factors can affect the quality of evidence and the strength of the recommendations made in the systematic review.

In summary, the GRADE approach is a rigorous method for evaluating the quality of evidence and developing recommendations based on the findings of a systematic review. By considering the study design, risk of bias, inconsistency, indirectness, and reporting bias, the GRADE approach provides a comprehensive assessment of the quality of evidence, which can help guide decision making and inform practice. 

Indirectness is an essential aspect of assessing the quality of evidence in systematic reviews. It refers to the degree to which the population, intervention, comparator, and study outcomes match the PICO question [18]. Indirectness was assessed based on the relevance and applicability of the study results to the PICO question. If a study did not address the PICO question directly or if the population, intervention, comparator, or outcomes substantially differed from those specified in the PICO question, the study was considered indirect.

Imprecision is another crucial aspect of assessing the quality of evidence. It refers to the degree of uncertainty in the results due to small sample sizes or wide confidence intervals [18]. Imprecision was assessed based on the precision and variability of the estimates across studies. Studies with small sample sizes or wide confidence intervals were considered imprecise and were assigned a lower evidence grade.

Reporting bias refers to the selective reporting of outcomes, such as publication or outcome reporting bias. Reporting bias was assessed based on the completeness and transparency of reporting the study results [18]. If a study did not report all relevant outcomes or selectively reported outcomes that favoured the intervention, it was considered to have a reporting bias. Such studies were assigned a lower grade of evidence, as they may overestimate the intervention’s effect size. The following table indicates the studies that were assessed for quality of evidence using the GRADE approach (see Table 2).

The overall quality of evidence indicates how confident we can be in the findings of the studies included in the systematic review. A high quality of evidence means we can have a high degree of confidence in the findings, while a low quality of evidence means less confidence.

#### 2.5.1. Knowledge and Understanding of Epilepsy

In this case, there is a high quality of evidence for knowledge and understanding of epilepsy based on one study, moderate quality was evident (*n* = 7), and only two studies showed low quality which was based on the small sample size of the selected studies. This means the possibility of improving knowledge and understanding of epilepsy by education intervention is moderate to high. Researchers can be confident that epilepsy education programs enhance knowledge and understanding of epilepsy among primary school learners and teachers.

#### 2.5.2. Values and Attitudes towards Epilepsy

According to GRADE, values and attitudes towards epilepsy and skills related to recognizing and responding to seizures were found to be mostly of moderate to low quality. This means that the degree of confidence in the findings is moderate. However, there are limitations to the evidence that needs to be considered. In the studies that were included, the limitations included the lack of a comparator.

#### 2.5.3. Skills Related to Recognizing and Responding to Seizures

The studies that were reviewed in the systematic review were graded, and the aspect of skills related to recognizing and responding to seizures was rated from moderate to low. Even though studies included in the systematic review were rated as moderate to low quality on skills related to recognizing and responding to seizures, there is a compelling rationale for including them in the analysis. Firstly, it is essential to acknowledge that studies with low ratings can still provide valuable insights and contribute to the overall evidence base [30]. Secondly, it is possible that including studies with lower ratings can help identify potential areas of weakness or inconsistency in the literature, which can inform future research efforts [31].

Overall, the quality of evidence suggests that there is some evidence to support the effectiveness of epilepsy education programs in improving knowledge and understanding of epilepsy, values and attitudes towards epilepsy, and skills related to recognizing and responding to seizures. Still, more high-quality research is needed to confirm these findings.

## 3. Results

### 3.1. Developing Recommendations

The systematic review that utilized the PICOS framework has yielded several key recommendations based on the evidence gathered from the included studies. These recommendations inform the development of epilepsy life skills guidelines.

❖ Epilepsy education should be incorporated into primary school curricula in life skills subjects to improve knowledge and understanding of epilepsy, values and attitudes towards epilepsy, and skills for recognizing and responding to seizures among primary school learners.
❖ Epilepsy education in life skills should be tailored to the needs of primary school learners. They should include information about epilepsy, seizure recognition and management, medication management, first aid, and coping strategies.
❖ Epilepsy education should be delivered in various settings, including classrooms, hospitals, and community centres, and by trained educators or healthcare providers.
❖ Healthcare providers, educators, and policymakers should work together to develop and implement epilepsy life skills education that is evidence-based and culturally appropriate.
❖ Future research should identify the most effective content, duration, and delivery method for epilepsy education in life skills among primary school learners.
❖ Primary school teachers and healthcare providers should receive training on epilepsy and how to support students with epilepsy, including providing appropriate accommodations and support during seizures.
❖ Families of primary school learners with epilepsy should be involved in developing and implementing epilepsy education in life skills to ensure that it meets their needs and addresses their concerns.

The recommendations are consistent with the research outcomes conducted during phase 1: stages 1 and 2 of the primary study [14,19,20]. These research stages aimed to explore the attitudes and viewpoints of different groups, including learners, teachers, and life skills advisors, regarding integrating epilepsy into life skills education.

### 3.2. Writing of Epilepsy Life Skills Guidelines

Incorporation of Epilepsy Education into Primary School Curricula in life skills:

Education specialists and Department of Basic Education (DBE) policymakers should ensure that epilepsy education is incorporated into the school curricula in life skill subjects. This will help to improve knowledge and understanding of epilepsy, values and attitudes towards epilepsy, and skills related to recognizing and responding to seizures among primary school learners.

Actions to fulfil the recommendation:
  ❖ Education specialists, DBE policymakers, and other stakeholders should work together to develop guidelines that provide clear recommendations and guidance for integrating epilepsy education into primary school curricula in life skills. These guidelines should outline the objectives of epilepsy education in life skills, the age-appropriate content, and the necessary resources for implementation.
  ❖ DBE and other stakeholders should allocate resources and support schools in implementing the incorporation of epilepsy education in life skills. These resources may include funding, teacher development, and necessary materials and equipment.
  ❖ To ensure the successful achievement of objectives and the effective enhancement of knowledge, attitudes, and skills related to epilepsy, it is imperative for DBE, other stakeholders, and policy makers to actively monitor and evaluate the implementation of integrating epilepsy education within the life skills curriculum in primary schools.

Tailoring of Epilepsy education in Life Skills:

Epilepsy education in life skills should be tailored to the needs of primary school learners. This education should include information about epilepsy, seizure recognition and management, medication management, first aid, and coping strategies.

Actions to fulfil the recommendation:
  ❖ Education specialists, DBE policymakers, and other stakeholders should develop age-appropriate content for epilepsy education in life skills. This content should be tailored to primary school learners’ cognitive abilities and developmental stages.
  ❖ Epilepsy education in life skills should be inclusive of the cultural and linguistic diversity of primary school learners. Educators should be aware of their learners’ cultural and linguistic backgrounds and incorporate this knowledge into the design and delivery of the epilepsy education. Moreover, this curriculum should be inclusive and embrace learners with disabilities.
  ❖ Educators and healthcare providers should receive training on how to tailor epilepsy education in life skills to meet the needs of primary school learners. This training should focus on the development of culturally appropriate and inclusive content, the use of appropriate teaching strategies, and the use of proper language.

Delivery of Epilepsy Education in Life Skills:

Epilepsy education in life skills should be delivered in various settings, including classrooms, hospitals, and community centres using different teaching methods. Trained educators and/or healthcare providers should deliver this education.

Actions to fulfil the recommendation:
  ❖ Educators and healthcare providers should develop various appropriate inclusive teaching strategies for epilepsy life skills education (leaving no one behind). These methods can include classroom-based teaching, interactive workshops, online learning, and community-based education. The other teaching methods may be the use of drama, video, and simulation.
  ❖ Educators and healthcare providers in the context of epilepsy education should include the incorporation of Ubuntu philosophical values, such as honesty, trust, compassion, kindness, respect, and love, into their teachings. The ultimate goal of this approach would be to cultivate a culture of acceptance, empathy, and care among primary school learners.
  ❖ Educators and healthcare providers should receive training on delivering epilepsy life skills education effectively in different settings. This training should focus on developing appropriate teaching strategies for different settings, using appropriate technology, and managing different types of learners.
  ❖ Educators and healthcare providers should train the community about epilepsy and encourage community participation in delivering epilepsy life skills education. This community participation can include the involvement of community-based organizations, local leaders, traditional healers, and people with epilepsy who can provide personal experiences and insights to the learners.

Development of Evidence-Based and Culturally Appropriate Education:

Healthcare providers, educators, and DBE policymakers should work together to develop and implement epilepsy education in life skills that is evidence-based and culturally appropriate.

Actions to fulfil the recommendation:
  ❖ Researchers should conduct studies to evaluate the effectiveness of epilepsy education in life skills. This research can include randomized controlled trials, pre- and post-education evaluations, and qualitative studies.
  ❖ Healthcare providers, educators and policymakers should consult with community members and stakeholders to ensure that epilepsy education is culturally appropriate. This can include engaging with people with epilepsy, families affected, and community organizations to gather input and feedback on the programs.
  ❖ Healthcare providers, educators, and DBE policymakers should establish partnerships to develop and implement evidence-based epilepsy education. These partnerships can facilitate the sharing of knowledge, resources, and expertise to ensure that epilepsy education is effective and sustainable.

Future Research on Epilepsy Education:

Future research should identify the most effective content, duration, and delivery method for epilepsy education in life skills among primary school learners.

Actions to fulfil the recommendation:
  ❖ It is crucial for funding agencies to allocate sufficient resources to support research endeavours focused on epilepsy education in life skills. This funding plays a pivotal role in facilitating the evaluation of the effectiveness of incorporating epilepsy education within the context of life skills.
  ❖ Universities, healthcare providers, educators, DBE policymakers, and funding agencies should collaborate with other stakeholders to identify research gaps and priorities. This collaboration can facilitate the development of research agendas that address the most pressing questions related to epilepsy education in life skills.
  ❖ Researchers and other stakeholders should disseminate research findings to inform the development and implementation of epilepsy education in life skills. This can include publishing articles in academic journals, presenting findings at conferences, and sharing information through online platforms and social media.

Training for Primary School Teachers and Healthcare Providers:

Primary school teachers and healthcare providers should receive training on epilepsy education and how to support learners with epilepsy, including providing appropriate accommodations and support during seizures.

Actions to fulfil the recommendation:
  ❖ Healthcare specialists, the Department of Social Development, epilepsy specialists, and education specialist should develop training programs for primary school teachers and other healthcare providers on epilepsy. This training should cover the basics of epilepsy, seizure recognition and management, medication management, first aid and how to accept learners with epilepsy.
  ❖ The training program should encompass the provision of guidance to educators on how to appropriately request exam exemptions and make necessary accommodations for students with epilepsy who may experience seizure episodes during examinations.
  ❖ Accreditation or certification should be granted to primary school teachers and healthcare providers who have undergone specialized training on epilepsy, demonstrating their knowledge and proficiency in dealing with this condition.
  ❖ Primary school teachers and healthcare providers should receive continuous professional development opportunities to ensure that they are up to date on the latest research and best practices related to epilepsy management.
  ❖ Primary school management teams and DBE policymakers should encourage schools to implement epilepsy management plans. These plans should include guidelines for responding to seizures, ensuring that appropriate support is available to learners with epilepsy.

Involvement of Families in epilepsy education Development and Implementation:

Families of primary school learners with epilepsy should be involved in developing and implementing epilepsy education in life skills to ensure that it meets their needs and addresses their concerns.

Actions to fulfil the recommendation:
  ❖ Educators should establish partnerships with families of primary school learners with epilepsy. This can include engaging with families to gather input and feedback on epilepsy education in life skills.
  ❖ Educators should consult with families to identify their needs and concerns related to epilepsy education in life skills. This can include soliciting feedback on the content and delivery methods.

## 4. Validation of the Developed Guidelines

Validation is the process of ensuring that a guideline is accurate, reliable, and based on strong evidence. The rationale for undertaking validation is to ensure that the guideline is trustworthy and the recommendations are safe and effective for the target population. The process of validation typically involves reviewing the quality of evidence and ensuring that the recommendations are based on the best available evidence.

WHO [18] emphasizes the importance of reviewing developed guidelines by experts before their implementation to ensure that they are feasible, evidence-based, and applicable to the target population. Additionally, the review process should be transparent to assess the guidelines’ quality and identify any gaps before their implementation [18].

To achieve this, the researcher employed the e-Delphi technique to conduct validation because it is a structured communication method that seeks to achieve a convergence of opinions among a group of experts and was used to reach a consensus among the group members [32]. The e-Delphi technique was selected due to its capacity to gather individual perspectives on the guidelines without being influenced by other opinions, as would occur in a group setting using an online platform.

### 4.1. Sampling Technique

The Delphi technique serves to enhance the comprehensibility and substantiation of outcomes obtained through surveys, focus groups, and interviews [32]. Therefore, a group of experts was selected using purposive sampling and it included primary school teachers, special school teachers, educational specialists, curriculum advisors, people living with epilepsy, health promotion officers, and caregivers of people with epilepsy. These experts, totaling 14, represented the provinces of Mpumalanga and Limpopo. For equal representation, effort was made to have seven experts for each province.

### 4.2. Data Collection

Upon the conclusion of guideline development, supervisors conducted an assessment of the guidelines and the researcher employed telephonic communication to engage specific experts. The purpose was to elucidate the study’s objectives, disseminate the results obtained from the empirical phase, solicit consent from the experts to partake in the validation process and apprise them of the anticipated contributions. Subsequently, via email, each expert received the finalized guideline, a consent form, and an open-ended, self-administered questionnaire (See Table 3 which shows the data collection tool).

The primary objective of the validation process was to determine whether the guidelines were clearly and precisely developed, feasible, evidence-based, and free from ambiguous words. After the presentation, each expert was given an open-ended questionnaire to rate the guidelines independently without influencing the opinions of others. The experts were given a week to review the guidelines and send the questionnaire of the first round back.

### 4.3. Analysis

#### 4.3.1. Demographic Characteristics

Fourteen experts from Limpopo and Mpumalanga provinces participated in guideline validation. Out of the 14 experts, 9 were female, while the remaining 5 were male. The educational background of these experts varied, encompassing both degrees and diplomas. In terms of age, the experts ranged from 25 to 64 years old. For a detailed overview of the demographic characteristics of the experts involved, please refer to Table 4.

#### 4.3.2. Round One Analysis

Upon receiving the responses from the experts, a descriptive analysis was performed on the gathered data. All participants who agreed to take part in the validation process provided responses to the questionnaires, ensuring a complete dataset for analysis. The qualitative aspect of the analysis focused on evaluating the guidelines in terms of accuracy, clarity, relevance, comprehensiveness, flexibility, and acceptability.

The results indicated that all participants (100%) perceived the guidelines to be accurate, comprehensive, flexible enough to accommodate different educational settings, and generally acceptable. Among the 14 participants, 9 individuals specifically emphasized the relevance of incorporating the principles of UBUNTU, such as love, kindness, compassion, and empathy, into the provision of epilepsy education in primary schools. During the general assessment of the guidelines, the analysis revealed that the guidelines were well developed overall and unambiguous. Below is the table showing guideline validation results (see Table 5). 

#### 4.3.3. Round Two Analysis

To ensure consensus, the guidelines were amended to include the incorporation of *UBUNTU* principles and a second round of questionnaires was distributed among the experts together with the amended guidelines. The results showed that the guidelines were feasible and unambiguous, and the expert group agreed on their applicability to the target population. The transparent validation process ensured the quality of the guidelines, and their implementation was likely to be successful.

## 5. Discussion

The discussion highlights guidelines for incorporating epilepsy life skills education programs into primary school curricula. Key recommendations include developing age-appropriate content tailored to the needs of primary school learners, delivering programs in various settings, involving families in program development and implementation, and providing training for primary school teachers and healthcare providers on epilepsy and how to support students with epilepsy. These recommendations are consistent with the findings of other studies conducted in the field of epilepsy education. Eze [25] and Goel [12] found that incorporating epilepsy education into school curricula can improve knowledge and attitudes toward epilepsy among students. Another study by Nevin et al. [33] emphasized the importance of tailoring epilepsy education to the needs of learners, including information on seizure recognition and management, medication management, and coping strategies.

Similarly, studies by England et al. [34] and Musekwa et al. [35] highlighted the need for delivering epilepsy education in various settings, such as classrooms, hospitals, and community centers and involving trained educators or healthcare providers. The study also emphasized the importance of collaboration between healthcare providers, educators, and policymakers in developing and implementing epilepsy education.

In terms of the validation process, this study employed the e-Delphi technique to ensure the accuracy and reliability of the developed guidelines. This approach is consistent with the recommendations of the World Health Organization, WHO [18], which emphasizes the importance of involving experts in the review and validation of guidelines before their implementation.

The results of the validation process indicated a high level of agreement among the experts regarding the accuracy, clarity, relevance, comprehensiveness, flexibility, acceptability, and overall quality of the guidelines. This suggests that the guidelines developed in this study are trustworthy and can be considered safe and effective for primary schools.

The developed guidelines for the inclusion of epilepsy in life skills education in primary schools are supported by the findings of other studies. The validation process using the e-Delphi technique confirmed the accuracy, clarity, relevance, comprehensiveness, flexibility, acceptability, and overall quality of the guidelines. These guidelines provide valuable insights for policymakers, educators, epilepsy life skills advisors, and healthcare providers involved in epilepsy education in primary schools. The guidelines emphasize the importance of developing evidence-based and culturally appropriate programs and involving families. Further research and collaboration among stakeholders are necessary to ensure the effective implementation and continuous improvement of epilepsy education programs. Implementing these guidelines can improve knowledge, attitudes, and skills related to epilepsy among primary school learners, and future research should focus on evaluating the long-term impact of these guidelines.

## 6. Limitations of the Study

Limited generalizability: the study was conducted in the Limpopo and Mpumalanga provinces of South Africa, which may limit its generalizability to other regions or countries with different socio-cultural contexts.

Moderate to low-quality studies: the systematic review that followed PICO found that some of the studies included in the review were of moderate to low quality according to the GRADE system, which may affect the validity and reliability of the evidence base for the guidelines.

Lack of long-term evaluation: The study did not include a long-term evaluation of the implementation and impact of the guidelines on the knowledge, attitudes, and behaviours of primary school learners and teachers regarding epilepsy life skills.

## 7. Conclusions

By implementing these life skills guidelines, primary school learners with epilepsy will have improved knowledge and understanding of epilepsy, values and attitudes towards epilepsy, and skills related to recognizing and responding to seizures. Additionally, this will create a supportive environment for learners with epilepsy, enabling them to reach their full potential.

## Figures and Tables

**Table 1:** Preliminary results.

Stage 1 Results (Teacher Perceptions) Makhado et al. [19]
Participant’s knowledge about epilepsy
Participant’s perspectives regarding the importance of epilepsy inclusion in life skills education
Participant’s suggested method of teaching epilepsy in life skills education
Participant perspectives regarding information to be included in the epilepsy life skills program
**Stage 1 Results (Life skills educational advisor perceptions) Makhado et al. [20]**
Life skills educational advisors’ knowledge of epilepsy
Benefits of including epilepsy in life skills education
Content for epilepsy education
Methods of teaching epilepsy in life skills education
**Stage 2 Results (Learner perceptions) Makhado et al.** [14]
Learners’ reasons for the integration of epilepsy education into life skills training
Learners’ recommended teaching methods for epilepsy in life skills education

**Table 2:** GRADE (grading of recommendations, assessment, development, and evaluation) for articles included in the systematic review.

Studies Included in the Systematic Review	Knowledge and Understanding of Epilepsy	Values and Attitudes Towards Epilepsy	Skills Related to Recognizing and Responding to Seizures
Aldraje et al. [27]	Low quality	Low quality	Low quality
Martiniuk et al. [22]	High quality	Moderate quality	Moderate quality
Abou Khaled et al. [23]	Moderate quality	Moderate quality	Low quality
Brabcová et al. [25]	Moderate quality	Moderate quality	Low quality
Mecarelli et al. [28]	Moderate quality	Moderate quality	Low quality
Mecarelli et al. [29]	Moderate quality	Moderate quality	Low quality
Eze et al. [26]	Moderate quality	Low quality	Low quality
Goel et al. [12]	Moderate quality	Moderate quality	Low quality
Brabcova et al. [24]	Low quality	Moderate quality	Low quality

**Table 3:** Data collection tool.

Questionnaire: Validation of Guidelines							
We value your subjective opinion regarding the developed guidelines. Please answer each question in the questionnaire based on your assessment of accuracy, clarity, relevance, comprehensiveness, flexibility, acceptability, and overall quality.							
Section A: Accuracy							
Question	YES	NO	Comment				
To what extent do you believe the guidelines accurately reflect current knowledge and evidence in epilepsy education? Rate from 1–5 and substantiate.			1	2	3	4	5

2. Are there any specific statements or recommendations that you find inaccurate or unsupported by evidence? Please provide details.							
Section B: Clarity							
How clear and understandable are the guidelines in terms of language and presentation?							
2. Are there any areas or statements that you find confusing or unclear? If yes, please provide examples.							
3. Is the guideline free from ambiguity?							
Section C: Relevance							
Do you believe that the guidelines address the important aspects and challenges of epilepsy education in primary schools?							
2. Are the guidelines evidence-based?							
3. Are there any crucial areas or topics that you think should be included but are missing from the guidelines? Please elaborate.							
Section D: Comprehesiveness							
Do you feel that the guidelines cover a wide range of relevant topics and considerations related to epilepsy life skills education?							
2. Are there any specific areas that you believe should be further expanded or elaborated upon?							
Section E: Flexibility							
Do you think the guidelines offer enough flexibility to accommodate different educational settings and diverse learner needs?							
2. Are there any suggestions you have to enhance the flexibility of the guidelines?							
Section F: Acceptability							
How acceptable do you find the guidelines to be in terms of feasibility and practicality for implementation in primary schools?							
2. Are there any recommendations that you consider impractical or difficult to implement? Please provide examples.							
Section G: General Assessment							
On a scale of 1 to 5, how would you rate the overall quality and usefulness of the guidelines?			1	2	3	4	5

2. Do you have any general comments or feedback regarding the guidelines that have not been covered in the previous questions?							

**Table 4:** Demographic characteristics.

Demographics		Frequency (*n* = 14)	Percent
**Gender**	Male	5	36%
	Female	9	64%
**Age**	25–30	2	14%
	31–49	8	57%
	50–64	4	29%
**Level of Education**	Degree/	5	36%
	Diploma	9	64%
**Job Title**	Caregivers of PLWE	2	14.3%
	Teachers	3	21.4%
	Curriculum advisors	1	7.1%
	Special school teachers	4	28.6%
	Educational specialist	1	7.1%
	Health promotion officers	2	14.3%
	PLWE	1	7.1%
**Total**		14	100%

**Table 5:** Guideline validation results.

Evaluation Criteria	Percentage of Agreement (%)	Findings and Observations
**Accuracy**	100%	All participants agreed that the guidelines were accurate.
**Clarity**	100%	All participants agreed that the guidelines were clear.
**Relevance**	64%	Nine participants emphasized the importance of incorporating UBUNTU principles in epilepsy education.
**Comprehensiveness**	100%	All participants agreed that the guidelines were comprehensive.
**Flexibility**	100%	All participants agreed that the guidelines were flexible to accommodate different educational settings.
**Acceptability**	100%	All participants found the guidelines to be acceptable.
**General Assessment**	100%	All participants unanimously gave the guidelines the highest rating of 4 out of 5 in terms of overall quality and usefulness.
		The guidelines were overall well developed and unambiguous.

## Data Availability

Since no new data were generated or analyzed for this study, the data sharing policy does not apply to this article.
